# Effects of computer monitor-emitted radiation on oxidant/antioxidant balance in cornea and lens from rats

**Published:** 2009-12-02

**Authors:** Mehmet Balci, Mehmet Namuslu, Erdinç Devrim, İlker Durak

**Affiliations:** 1 Dr. Abdurrahman Yurtaslan Oncology Training and Research Hospital, Department of Ophthalmology, Ankara, Turkey; 2 Ankara University, Faculty of Medicine, Department of Biochemistry, Ankara, Turkey

## Abstract

**Purpose:**

This study aims to investigate the possible effects of computer monitor-emitted radiation on the oxidant/antioxidant balance in corneal and lens tissues and to observe any protective effects of vitamin C (vit C).

**Methods:**

Four groups (PC monitor, PC monitor plus vitamin C, vitamin C, and control) each consisting of ten Wistar rats were studied. The study lasted for three weeks. Vitamin C was administered in oral doses of 250 mg/kg/day. The computer and computer plus vitamin C groups were exposed to computer monitors while the other groups were not. Malondialdehyde (MDA) levels and superoxide dismutase (SOD), glutathione peroxidase (GSH-Px), and catalase (CAT) activities were measured in corneal and lens tissues of the rats.

**Results:**

In corneal tissue, MDA levels and CAT activity were found to increase in the computer group compared with the control group. In the computer plus vitamin C group, MDA level, SOD, and GSH-Px activities were higher and CAT activity lower than those in the computer and control groups. Regarding lens tissue, in the computer group, MDA levels and GSH-Px activity were found to increase, as compared to the control and computer plus vitamin C groups, and SOD activity was higher than that of the control group. In the computer plus vitamin C group, SOD activity was found to be higher and CAT activity to be lower than those in the control group.

**Conclusion:**

The results of this study suggest that computer-monitor radiation leads to oxidative stress in the corneal and lens tissues, and that vitamin C may prevent oxidative effects in the lens.

## Introduction

An increasing number of people report subjective symptoms and hypersensitivity to a wide variety of electromagnetic sources including power lines, radio and TV broadcasting stations, cellular phones, and computer monitors [1]. A personal computer (PC) is standard equipment for many people in industrialized societies, and its use is growing. A PC user is exposed to an electromagnetic field (EMF), visible and ultraviolet light, radio-range waves, and extremely low frequency (50 Hz) fields (ELF) [2]. Preliminary experiments showed that radiation from a monitor can produce potentially hazardous biological effects [2].

There is increasing interest in the potential health risks and biologic effects related to exposure to ELF-EMFs. Among the environmental risk factors that affect human health, ELF-EMFs play an important role because of their possible association with childhood malignancy, especially leukemia, but also cancer and cardiovascular, neurological, and psychological diseases in adults [3-6]. The incidence of premature births and infants born with pathologies and the risk of brain tumors are higher for PC users [7]. PC monitor radiation stimulates the growth of urethane-induced lung tumors in mice [8]. Although eye-related effects of PC monitor use have not yet been discovered, past studies have demonstrated a relation between electromagnetic radiation (EMR) and cataracts [9]. Corneal and retinal damage has been observed as well [10,11].

Much research interest has emerged about the mechanisms of the interaction between ELF-EMFs and living organisms. Experimental studies have shown that ELF-EMFs may interfere with chemical reactions involving free radical production [12-14]. Moreover, some researchers have reported that PC monitor use is associated with the free radical process [2,15]. The eye is an exceptional organ because of its continuous exposure to environmental chemicals, radiation, and atmospheric oxygen. Oxidative stress mechanisms in ocular tissues have been hypothesized to play a role in diseases such as cataracts, glaucoma, uveitis, pseudoexfoliation syndrome, and age-related macular degeneration [16,17].

Vitamin C (vit C), a powerful antioxidant, provides a protective effect against several diseases, including oxidative imbalances arising from various causes in the lens [18-20]. At the same time, vit C acts as a pro-oxidant, depending upon the environment in which the molecule is present. It has been reported that millimolar concentrations of vit C induced apoptotic cell death, characterized by cell shrinkage, nuclear fragmentation, and internucleosomal DNA cleavage in human myelogenous leukemic cell lines. Higher concentrations of vit C induce apoptotic cell death in various tumor cell lines, including oral squamous cell carcinoma and salivary gland tumor cell lines, possibly via its pro-oxidant action. The apoptosis-inducing activity of vit C is stimulated by Cu^2+^, lignin, and ion chelator and inhibited by catalase, Fe^3+^, and Co^2+^. As far as we know, there is no study in the existing literature investigating the possible protective effects of vit C against PC monitor radiation in the corneal and lens tissues [20-25].

The aim of the present study is to investigate the effects of PC monitor-emitted radiation on oxidative stress and antioxidant enzyme activities in the corneal and lens tissues of rats and to observe any protective effects of vitamin C.

## Methods

Forty female Wistar albino type rats (average 160 g and six weeks old, in puberty period) were used in the study. The study was approved by the Ethics Committee of Ankara Oncology Hospital. The animals received appropriate animal care, and the study protocol complied with the institution’s guidelines of the Health Ministry. Four groups were constituted, and in each group (control, PC monitor, PC monitor plus vitamin C, and vitamin C) there were ten animals. The PC monitor group was exposed to two cathode ray tube (CRT) type monitors positioned face-to-face, approximately 20 cm away from the cages. Brightness/contrast settings were adjusted to 50% of the maximum levels of each.

The PC monitor plus vitamin C group was exposed to the same monitors and received vitamin C in solution form, in oral doses of 250 mg kg^−1^ day^−1^ [26]. The vitamin C group was treated with vitamin C only, in the same manner. The PC monitor groups were exposed to an EMF for 8 h/day, every day, for a period of three weeks. The rats were group-caged and were allowed free motion in their cages. The control and vitamin C groups were in another room in which there was no EMF. Throughout the study period, these two groups were never exposed to an EMF.

During the study, all the animals, including the control group, were fed a laboratory diet and water ad libitum. At the end of the study period, the animals were sacrificed under ether anesthesia and their corneal and lens tissues were surgically dissected. The tissues were homogenized and prepared for the assays as described previously [27]. The upper, clear part of the tissue homogenates (supernatants) was used in the measurements. The protein levels of the clear supernatants were studied using the Lowry method [28]. MDA levels (nmol/mg), SOD (U/mg), GSH-Px (mIU/mg), and CAT (IU/mg) enzyme activities were measured from the supernatants. MDA levels were measured by the thiobarbituric acid reactive substances (TBARS) method [29]. SOD activity was measured as described previously [30]. One unit for SOD activity was expressed as the enzyme protein amount causing 50% inhibition in the nitro blue tetrazolium (NBT) reduction rate. Catalase activity was determined by measuring the absorbance decrease of H_2_O_2_ at 240 nm [31]. GSH-Px activity was measured by following changes in nicotinamide adenine dinucleotide phosphate (NADPH) absorbance at 340 nm [32]. In the activity calculations, extinction coefficients of H_2_O_2_ (40.98 L mol^-1^ cm^-1^ at 240 nm) and NADPH (6220 L mol^-1^ cm^-1^ at 340 nm) were used for CAT and GSH-Px enzymes, respectively.

All statistical analyses were carried out using SPSS statistical software (SPSS for Windows, version 12.0, SPSS Inc., Chicago, IL). Data were given as arithmetic mean ±standard deviation (mean±SD). In the statistical evaluation of the results, the Student’s *t* test was carried out. Statistical significance was defined as a p value lower than 0.05.

## Results

The corneal and lens levels of the oxidative parameters for all groups are given in Table 1 and Table 2, respectively. In the corneal tissue, there were significant increases in the MDA level and CAT activity in the PC monitor group, compared with the control group (p<0.05), whereas there was no difference in SOD and GSH-Px activities between the two groups. In the PC monitor plus vitamin C group, the MDA level, SOD, and GSH-Px activities were significantly higher, and CAT activity was significantly lower, compared with the corresponding values in the PC monitor group (p<0.05). In the PC monitor plus vitamin C group, the MDA level, SOD, and GSH-Px activities were significantly higher (p<0.05), and CAT activity was significantly lower (p<0.05) than in the control group.

**Table 1:** PC monitor effects in the corneal tissue (Oxidant/antioxidant parameters, Mean±SD).

** Groups**	**MDA (nmol/mg)**	**SOD (U/mg)**	**GSH-Px (mIU/mg)**	**CAT (IU/mg)**
PC monitor	0.323±0.021*	26.46±7.74	45.59±33.63	12.03±1.08*
PC monitor + Vit C	0.439±0.098**,#	34.71±7.88**,#	101.18±3.73**,#	1.84±1.65**,#
Vit C	0.270±0.014	17.95±4.90	26.86±3.65	2.39±1.09
Control	0.254±0.012	22.10±6.94	39.22±5.11	4.52±2.06

The asterisk indicates a p<0.05 (Student’s t test) for the PC monitor group versus the Control group. The double asterisk indicates a p<0.05 (Student’s t test) for the PC monitor plus vitamin C group versus the PC monitor group. The sharp (hash mark) indicates a p<0.05 (Student’s t test) for the PC monitor plus vitamin C group versus the Control group.

**Table 2:** PC monitor effects in the lens tissue (Oxidant/antioxidant parameters, Mean±SD).

** Groups**	**MDA (nmol/mg)**	**SOD (U/mg)**	**GSH-Px (mIU/mg)**	**CAT (IU/mg)**
PC monitor	0.058±0.023*	4.91±1.75*	7.51±1.18*	0.580±0.525
PC monitor + Vit C	0.035±0.004**	3.90±0.42#	5.48±1.88**	0.435±0.077#
Vit C	0.034±0.003	3.47±0.06	6.18±0.20	0.934±0.060
Control	0.036±0.008	3.41±0.36	6.03±0.61	0.565±0.069

The asterisk indicates a p<0.05 (Student’s t test) for the PC monitor group versus the Control group. The double asterisk indicates a p<0.05 (Student’s t test) for the PC monitor plus vitamin C group versus the PC monitor group. The sharp (hash mark) indicates a p<0.05 (Student’s t test) for the PC monitor plus vitamin C group versus the Control group.

In the results obtained for lens tissue, the MDA level, SOD, and GSH-Px activities were confirmed to be significantly increased in the PC monitor group relative to the control group (p<0.05). As for CAT activity, no significant difference was found between the two groups. When the PC monitor group was compared with the PC monitor plus vitamin C group, significantly lower MDA levels and GSH-Px activity were found in the latter group (p<0.05). No significant differences were seen between the two groups in terms of SOD and CAT activities. In the PC monitor plus vitamin C group, SOD activity was significantly higher (p<0.05), and CAT activity was significantly lower (p<0.05) than in the control group.

## Discussion

In order to explain the epidemiological observations associated with ELF-EMF exposure, experiments have been conducted in multiple laboratories to examine alterations of biological functions by EMF at the cellular and molecular levels. Cellular studies have described a variety of EMF effects on biological and biochemical responses, including cell proliferation [33,34], cell surface properties [35], apoptosis induction [36], and DNA damage [37]. Among the putative mechanisms, ELF-EMFs may affect biological systems by increasing free radical life span and the concentration of free radicals (or other reactive oxygen species - ROS) in cells [12,38-41].

It is well known that ROS lead to oxidative damage in major cell macromolecules, such as lipids and nucleic acids. ROS have been implicated in tissue injury. The main ROS that have to be considered are the superoxide anion (O_2_**^-.^**), which is predominantly generated by the mitochondria; H_2_O_2_ produced from O_2_**^-.^** by the action of SOD; and peroxynitrite generated by the reaction of O_2_**^-.^** with nitric oxide. ROS are scavenged by SOD, GSH-Px, and CAT. MDA is the breakdown product of the major chain reactions leading to the oxidation of polyunsaturated fatty acids and, thus, serves as a reliable marker of oxidative stress-mediated lipid peroxidation [42].

The disruption of the oxidant/antioxidant balance in the eye and other tissues exposed to EMR from mobile phones has been shown in experimental studies [11,43]. In addition, we found that mobile phone radiation leads to oxidative stress due to increased MDA levels in the cornea and lens [44]. Falone et al. [45] indicated that ELF-EMF exposure significantly affects anti-oxidative capability, and they suggested that exposure to ELF-EMFs may act as a risk factor for the occurrence of oxidative stress-based nervous system pathologies. Moreover, some researchers have recently linked the role of ELF-EMFs in activating immune-relevant cell types to the free radical-based physiological changes detected following field exposure [46,47].

Yokus et al. [38] reported increased lipid peroxidation oxidative DNA damage in rats exposed to ELF-EMFs. Furthermore, Guler et al. [48] found a significant increase in the levels of MDA and a significant decrease in antioxidant enzyme activities in Guinea pigs that were exposed to an ELF-electric field. They also indicated that N-acetyl-L-cysteine application has protective effects on ELF-electric field-induced oxidative stress. In the present study, we detected clear changes due to oxidative stress in the cornea, in accordance with previous studies. In corneas exposed to PC monitor radiation, the MDA level, as an indicator of lipid peroxidation, significantly increased. The cornea, being lipid-rich tissue, may manifest this marked increase in MDA [49].

A number of studies have been performed to evaluate the antioxidant effects on EMF-induced oxidative damage [11,43]. We also investigated the effectiveness of a powerful antioxidant, vitamin C, on the oxidative damage induced by monitor radiation under the present experimental conditions. Vitamin C treatment on the radiation-exposed groups resulted in significantly increased SOD and GSH-Px activities. However, vitamin C could not protect corneal tissue against PC monitor radiation-induced oxidative stress, as revealed by increased MDA levels, and decreased catalase activity, in the PC monitor plus vitamin C group compared to the PC monitor group.

In the lens tissue, significantly increased MDA levels, SOD, and GSH-Px activities were found in the group exposed to radiation compared to the control group. SOD and GSH-Px activities may increase as a compensatory mechanism to eliminate this oxidative stress. We observed a significant decrease in MDA levels in the lens tissue with the administration of vitamin C in the PC monitor group, compared to the PC monitor alone group. Additionally, SOD activity was higher in the PC monitor plus vitamin C group than in the control group.

In conclusion, exposure to PC monitor radiation may act as a risk factor for the occurrence of oxidative stress-based cornea and lens pathologies. The potent free radical scavenger and antioxidant, vitamin C, may protect lens tissues from oxidative damage, thus preventing organ dysfunction. Considering the widespread use of computers, it will be essential to evaluate the long-term effects of computer monitor radiation on the eye, as well as protective measures. There is a need for further study with different frequencies and exposure periods in order to discover the effects of PC monitor radiation-induced oxidative stress in the eye.
